# Supplementary material for: Refined methodology for quantifying Pseudomonas aeruginosa virulence using Galleria mellonella
Source: Microbiol Spectr. 2024 Dec 12;13(2):e01666-24. doi: 10.1128/spectrum.01666-24 (PMC11792518; doi:10.1128/spectrum.01666-24)
Supplement: Supplemental figures — Fig. S1 and S2. [file spectrum.01666-24-s0001.pdf]

Refined methodology for quantifying *Pseudomonas aeruginosa* virulence using *Galleria mellonella*

Christopher M. R. Axline, Travis J. Kochan Sophia Nozick, Timothy Ward, Tania Afzal, Issay Niki, Sumitra D. Mitra, Ethan VanGosen, Julia Nelson, Aliki Valdes, David Hynes, William Cheng, Joanne Lee, Prarthana Prashanth, Timothy L. Turner, Nathan B. Pincus, Marc H. Scheetz, Kelly E. R. Bachta, Alan R. Hauser

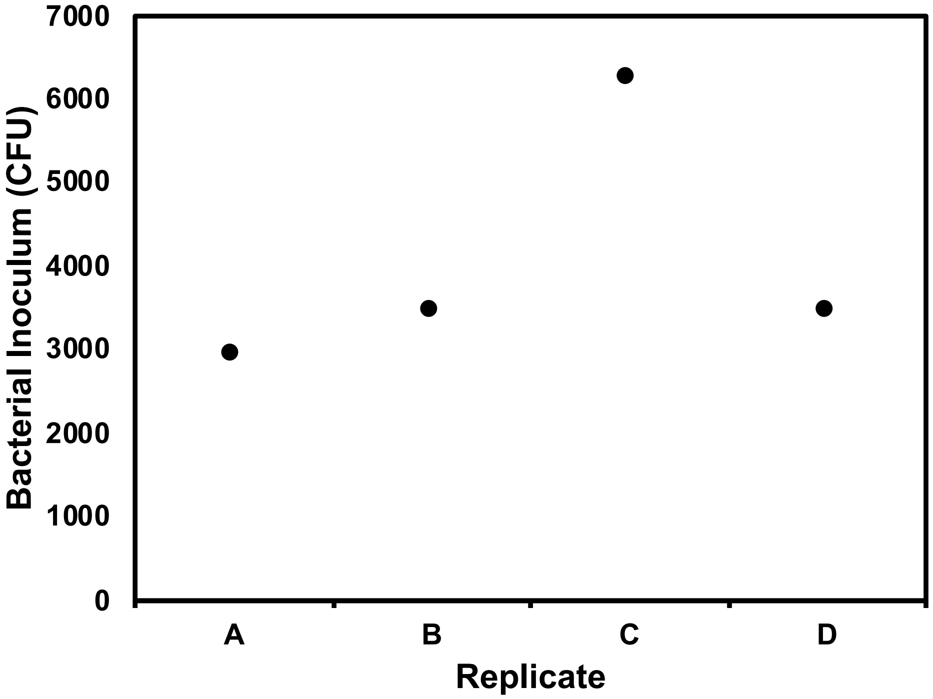

Supplemental Fig 1. Example of the difficulty in reproducibly preparing a small inoculum of *Pseudomonas aeruginosa*. A culture of *P. aeruginosa* strain PABL089 was split into four tubes (A, B, C, D). Each tube was independently adjusted to an OD_{600} of 0.2 and diluted appropriately to obtain an estimated inoculum of 2000 CFU/10 μl for injection into *G. mellonella*. To quantify the actual CFU in the inoculums, each sample was plated for colony enumeration.

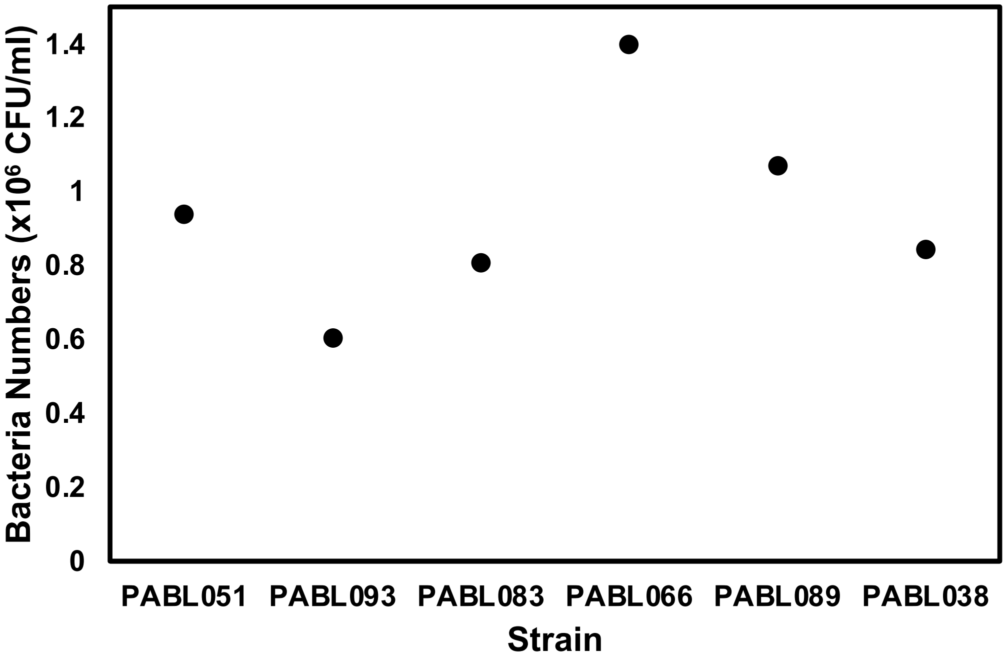

Supplemental Fig 2. *Pseudomonas aeruginosa* strain-to-strain variation in CFU/ml obtained from suspensions with the same OD_{600}. Six different *P. aeruginosa* strains (PABL051, PABL093, PABL083, PABL066, PABL089, and PABL038) were cultured and diluted to an OD_{600} of 0.20. CFU/ml values were then determined by dilution, plating, and colony enumeration.
