# Supplementary material for: Refined methodology for quantifying Pseudomonas aeruginosa virulence using Galleria mellonella
Source: Microbiol Spectr. 2024 Dec 12;13(2):e01666-24. doi: 10.1128/spectrum.01666-24 (PMC11792518; doi:10.1128/spectrum.01666-24)
Supplement: Supplemental methods — Protocol for performing Galleria experiments. [file spectrum.01666-24-s0002.pdf]

Refined methodology for quantifying *Pseudomonas aeruginosa* virulence using *Galleria mellonella*

Christopher M. R. Axline, Travis J. Kochan Sophia Nozick, Timothy Ward, Tania Afzal, Issay Niki, Sumitra D. Mitra, Ethan VanGosen, Julia Nelson, Aliki Valdes, David Hynes, William Cheng, Joanne Lee, Prarthana Prashanth, Timothy L. Turner, Nathan B. Pincus, Marc H. Scheetz, Kelly E. R. Bachta, Alan R. Hauser

Protocol for Measuring LT_{50} Values of *Pseudomonas aeruginosa* Strains Using *Galleria mellonella*

Preparation of Larvae

1. Sort larvae based on weight and appearance.
   a. Weight range: Weigh larvae and select those weighing 200-350 mg
   b. Appearance: Larvae should be devoid of dark coloration (Fig S1) and should readily move upon stimulation. Larvae not moving normally or with melanin spots should be discarded.

Protocol Fig 1. Appearance of healthy and unhealthy *G. mellonella* larvae. A. Healthy larva without pigmentation. B. Unhealthy larva with melanin spots (arrows).

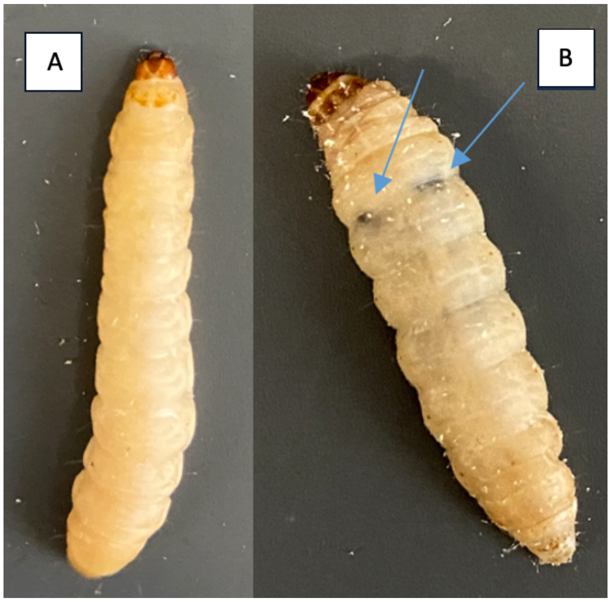

Preparation of Bacterial Inoculums

2. On Lysogeny broth (LB) agar plates, streak out desired *P. aeruginosa* strains from frozen stocks. Incubate at 37˚C overnight.

3. Pick a single colony and inoculate into 5 mL liquid LB media. Incubate overnight with shaking (250 RPM) at 37˚C.

4. The following morning, subculture 50 µL in 5 mL (1:100 dilution) of liquid LB media.

5. Incubate with shaking (250 RPM) at 37˚C for 3 hours (to an OD_{600} of ~0.5-0.8).

6. Centrifuge for 10 minutes at 3,200 × *g* at room temperature (21˚C), decant, and resuspend the pellet in 1 mL of sterile phosphate-buffered saline (PBS). Transfer to a 1.5 mL microcentrifuge tube.

7. Centrifuge at 17,000 × *g* for 2 min, decant, and resuspend the pellet in 1 mL PBS.

8. Adjust via dilution with PBS to an OD_{600} of ~0.2 (~5 × 10^7 CFU/ml). Note that the actual OD_{600} to CFU/mL relationship may vary substantially from strain to strain, so the actual number of CFU in the inoculum should be determined by plating and enumeration (see below).

9. Perform serial 10-fold dilutions in PBS as described in Table S1 to generate the desired doses for injection into *G. mellonella* larvae. Keep at room temperature (not on ice) and use immediately for larval injections and inoculum CFU enumerations (see step 14).

Table S1. Typical dilution series for target doses*

Target Dose<br><br>(CFU/10 µL)	Stock dilution	PBS (µL)	Bacterial suspension from previous tube (µL)
500,000	1		
50,000	10^{-1}	900	100
5,000	10^{-2}	900	100
500	10^{-3}	900	100
50	10^{-4}	900	100

*The range of target doses may need to be adjusted based on an individual strain's virulence.

Larval Injection

10. Set up the injection apparatus (Fig S2). Ensure that the bevel of the needle is facing upwards when clamped in the autoinjector

Syringes: EXELINT Comfort Point 0.5 mL Insulin Syringes (29G x ½")

Auto-Injector: KD Scientific Legato 100 (Catalog Number: 788100)

Protocol Fig 2. Larval injection system.

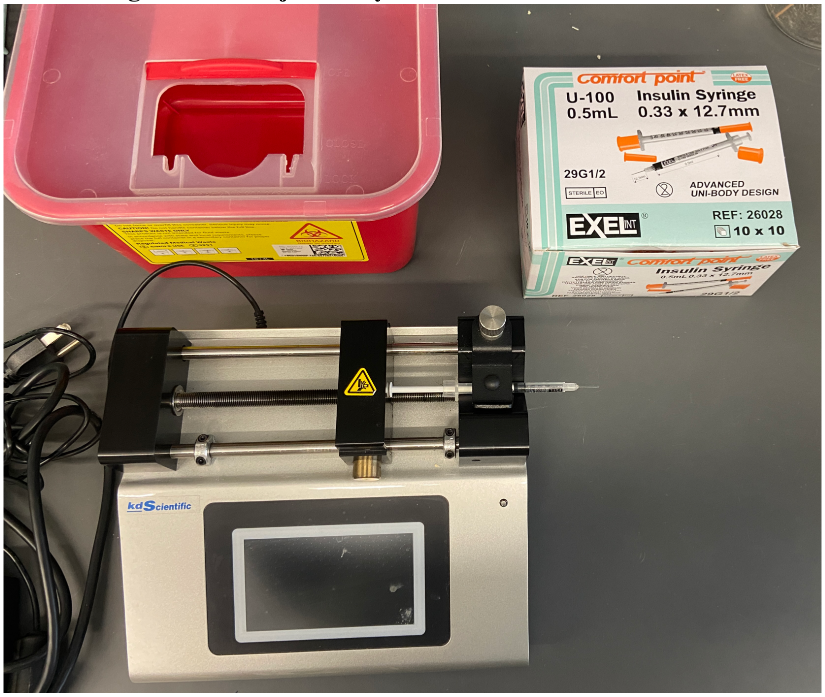

11. Fill syringe with one of the bacterial suspensions prepared in Table S1. Hold the larva against the syringe (Fig S3). Inject 10 µL into the final proleg of a larva (Fig S4). To enhance safety and prevent needlesticks, users may wish to use a sponge/clamp system (Dalton JP, Uy B, Swift S and Wiles S. 2017. A Novel Restraint Device for Injection of *Galleria mellonella* Larvae that Minimizes the Risk of Accidental Operator Needle Stick Injury. Front. Cell. Infect. Microbiol. 7:99. doi: 10.3389/fcimb.2017.00099). Place the larva into a Petri dish with the dose and strain marked on the dish.

Protocol Fig 3. Use of the Auto-Injector instrument to inoculate *G. mellonella.*

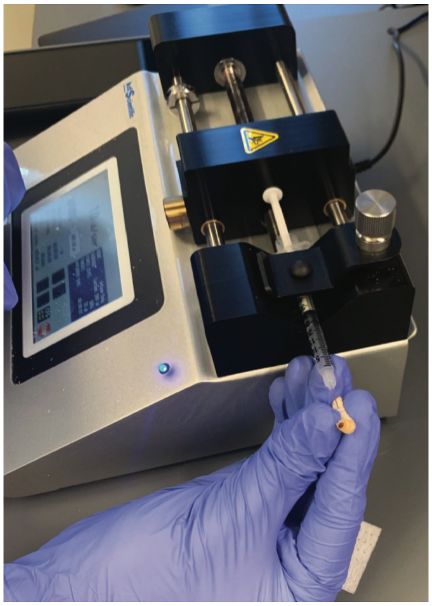

12. Repeat for a total of 10 larvae per dose per strain. Include a set of 10 larvae injected with PBS as a negative control.

13. Incubate infected larvae in Petri dishes at 37˚C.

Protocol Fig 4. The site of the final proleg of *G. mellonella* larva (indicated by arrow).

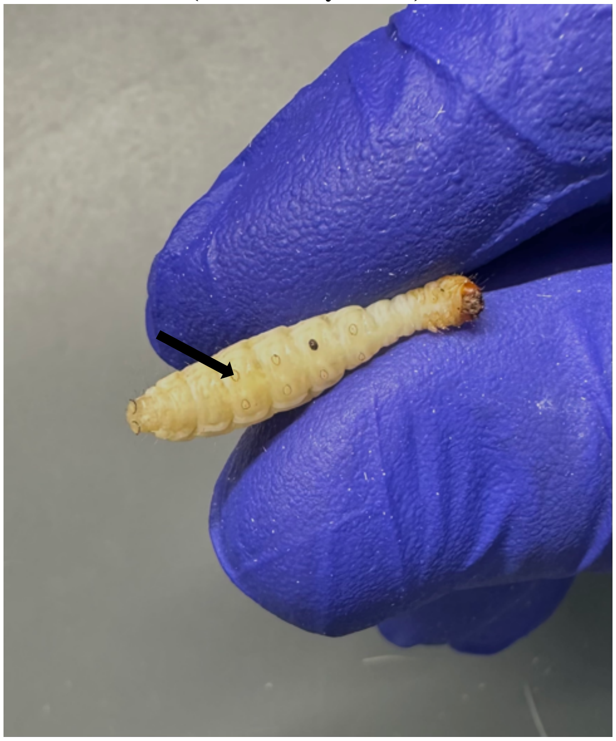

Enumeration of Bacterial Numbers in Inoculums

14. Plate serial dilutions of the remaining portions of the inoculum suspensions on LB agar plates to quantify the number of CFU in each inoculum. Incubate overnight at 37˚C.

15. Count colonies to determine the number of CFU in each inoculum. Enter the CFU injected into larvae for each experiment in a mortality table (see Table S2 for an example).

Monitor *G. mellonella* for mortality

16. Check the larvae hourly for death. Larvae are adjudicated as dead when they fail to move following gentle shaking of the Petri dish. Note that melanization is not an accurate indicator of larval death, as it may be observed in living larvae.

17. Record the time of each check and the cumulative number of dead larvae in the mortality table (see Table S2 for example).

Table S2. Sample mortality table*

Strain/dose	Time	Total Larvae	Mortalities	CFU
**BL83-A**	0	10	0	73000
**BL83-A**	5	10	0	73000
**BL83-A**	6	10	1	73000
**BL83-A**	7	10	1	73000
**BL83-A**	8	10	1	73000
**BL83-A**	9	10	1	73000
**BL83-A**	10	10	1	73000
**BL83-A**	11	10	8	73000
**BL83-A**	13	10	10	73000
**BL83-A**	14	10	10	73000
**BL83-B**	0	10	0	36500
**BL83-B**	5	10	0	36500
**BL83-B**	6	10	0	36500
**BL83-B**	7	10	0	36500
**BL83-B**	8	10	0	36500
**BL83-B**	9	10	0	36500
**BL83-B**	10	10	0	36500
**BL83-B**	11	10	10	36500
**BL83-B**	13	10	10	36500
**BL83-B**	14	10	10	36500

*Table must be .csv format

Estimation of LT_{50} Values

18. The final mortality table should be populated with the *P. aeruginosa* strain designation, the times at which the larvae were checked for mortality (in hours post-inoculation), the total number of larvae inoculated with the dose (usually 10), the cumulative number of dead

larvae at each time point, and the dose itself, as measured by plating and enumeration (in CFU). See Table S2 for an example of a completed mortality table.

19. Use the mortality table with the following R-script to estimate LT_{50} values: https://github.com/ChrisAxline/Galleria_Code.git. This script fits a sigmoidal curve to the mortality data to estimate the LT_{50} value for each dose.

Analysis and Quality Control

20. Plot the LT_{50} values vs. the natural log of the inoculum CFU for each strain.

21. Perform a linear regression on the data points for each strain to generate a line reflecting the LT_{50} – ln(CFU) relationship of each strain.

22. Perform quality control analysis by calculating the R^2 coefficient for the data points for each strain. Data points with substantial scatter (e.g., with low R^2 values) should be discarded, and the experiment repeated. (We use an R^2 value of 0.60 as a threshold for repeating an experiment)

23. The data to generate each LT_{50} – ln(CFU) plot should be independently generated at least twice to ensure reproducibility. Final results may be combined to generate a single LT_{50} – ln(CFU) curve.
